# Ultrasonography of Pregnancy in Murciano-Granadina Goat Breed: Fetal Growth Indices and Umbilical Artery Doppler Parameters

**DOI:** 10.3390/ani13040618

**Published:** 2023-02-09

**Authors:** David Ramírez-González, Ángel Poto, Begoña Peinado, Laura Almela, Sergio Navarro-Serna, Salvador Ruiz

**Affiliations:** 1 Department of Physiology, Faculty of Veterinary Medicine, Campus Mare Nostrum, University of Murcia, 30100 Murcia, Spain; 2 Murcian Institute of Agricultural and Environmental Research and Development, 30150 Murcia, Spain; 3 Institute for Biomedical Research of Murcia, IMIB-Arrixaca, 30120 Murcia, Spain

**Keywords:** Murciano-Granadina goat, pregnancy, Doppler ultrasonography, fetal growth, umbilical cord, blood flow

## Abstract

**Simple Summary:**

The evolution of some fetal growth indices (crown-rump length, trunk diameter, biparietal diameter and eye orbit diameter) and different blood flow parameters (arterial pulse, peak systolic velocity, end diastolic velocity, mean velocity, systolic velocity/diastolic velocity ratio and pulsatility and resistance indices) of the umbilical artery of the embryos and fetuses of primiparous pregnant goats of the Murciano-Granadina breed were analyzed by ultrasonography. Weekly ultrasonographic sessions took place from 18- to 125-days post-mating. Fetal measures were carried out by ultrasound B-mode. Spectral Doppler was used to study blood flow from umbilical artery. Umbilical cord was first noticed between 32- and 35-days post-mating. However, umbilical arterial blood flow parameters were not conclusive until 65–80 days of pregnancy. This is the first time that a detailed study of fetal growth indices and the umbilical artery blood flow rates in fetuses from Murciano-Granadina goats has been performed throughout virtually the entire duration of gestation, determining the evolution of these fetal growth parameters in this breed and how the velocimetric parameters are increasing significantly and the arterial pulse, systolic/diastolic ratio and pulsatility and resistance indices are decreasing significantly throughout the analyzed pregnancy period.

**Abstract:**

The evolution of some fetal growth indices and arterial blood flow parameters in the umbilical cord of the embryos and fetuses of primiparous pregnant goats of Murciano-Granadina breed were analyzed by ultrasonography. Weekly ultrasonographic sessions took place from 18- to 125-days post-breeding. Fetal measures were carried out by ultrasound B-mode. This mode was used to take a series of measurements in the embryo/fetus throughout pregnancy: crown-rump length (CRL, from 24-days post-mating -dpm- to 61 dpm), trunk diameter (TD, 24–34 dpm), biparietal diameter (BPD, 28–125 dpm) and eye orbit diameter (EOD, 75–125 dpm). Spectral Doppler was used to study blood flow from umbilical artery. Different blood flow parameters were obtained as follows: Arterial Pulse, Peak Systolic Velocity (PSV), End Diastolic Velocity (EDV), Mean Velocity (MV), Systolic velocity/Diastolic velocity Ratio (S/D), Pulsatility Index (PI) and Resistance Index (RI). In this study, the umbilical cord was first noticed between 32- and 35-days post-breeding. However, these umbilical arterial blood flow parameters were not conclusive (positive S/D ratios and RI < 1) until 65–80 days of pregnancy. The explanation to these results could be that vascular development related to umbilical arteries elasticity and diameter is not good enough in early pregnancy. Therefore, these vessels have already acquired their appropriate characteristics in order to allow blood flow parameters and Doppler index measures from only 2.5 months of pregnancy. This is the first time that a detailed study of fetal growth indices and umbilical artery flow rates in fetuses from Murciano-Granadina goats has been performed throughout virtually the entire duration of gestation. In conclusion, the evolution of the fetal growth indices in this breed has determined that the umbilical artery velocimetric parameters (PSV, EDV, MV) increase significantly and the AP, S/D, PI and RI indices decrease significantly throughout the analyzed pregnancy period.

## 1. Introduction

The Murciano-Granadina (M-G) is one of the most popular goat breeds in Spain. It has a great distribution abroad too, exporting animals to Europe, Africa and South America [1]. In Spain, the number census has been estimated around 500,000 animals that are distributed throughout the country, though mainly in Andalusia, Murcia and Castile-La Mancha [2]. This breed, which owes its name to the provinces of its origin in Spain (Murcia and Granada), is made up of dairy goats with a black or auburn coat and a height of about 70–77 cm [3]. It is easily adapted to different types of management and production, being able to develop optimally both in more intensive and technical systems as well as extensive and ecological ones [4]. On the other hand, animals of the M-G breed can be kept in production in very extreme conditions, from dry and hot climates to cold and mountainous ones [2].

Depending on the estrous cyclicity that they present, goats can be considered similar to seasonal polyestrous breeders with short days [5]. In this way, it is known that goats present their sexual activity in intervals of several heats (of 21 days) in a row, which take place at the time of year when there is a decreasing photoperiod. This reproductive activity changes depending on environmental and physiological factors (latitude, climate, food availability, breed and husbandry systems) [6]. However, the variations of the photoperiod are the most decisive influence on reproduction for this species. Thus, there are animals that, raised in areas whose latitude is closer to the equator, experience fewer changes in photoperiod and temperature, so they show a longer breeding season than those goats raised in temperate climates and polar regions [7]. Therefore, in the case of the M-G breed, there is no seasonal anestrus. Temperatures and photoperiod in the southern latitudes of the Iberian Peninsula (36–38° N) allow bucks to have a high libido, and goats to are able to react to them without the need for hormonal treatments to control their photoperiod [8].

For greater economic performance, farmers prefer to group the births of goats at the end of summer and in autumn, since this time coincides with the highest milk prices as well as the sale of kids between September and December, although it does not correspond to the reproductive seasonality of the goat [4]. The M-G breed generally gives birth once a year, having an average of 1.9 kids in each birth. It may range from one to two kids and can even reach up to five or six, although in this case, special care is required in goats. The first mating of goats usually occurs between six and eight months of age [4].

As previously mentioned, mating is usually scheduled to group births at the most favorable moment of the year from an economic point of view. For this, different reproductive methodologies are carried out for the synchronization of estrus in females, either non-hormonal such as the male or female effect (very marked in this species) or hormonal methods [4]. Vaginal devices impregnated with progestogens have been commonly used in hormonal synchronization. In addition of the traditional sponges, newer progestogen-releasing devices such as CIDR^®^ have been used, followed by injections of equine chorionic gonadotropin (eCG) [9]. Progestogens would have an inhibitory effect on the hypothalamic-pituitary-gonadal axis, and eCG would have a FSH-like activity, causing folliculogenesis in the ovary. Therefore, when progestogen is removed, there should be a well-developed follicle ready to ovulate [10]. Single or double injections of PGF2α have also been employed to induce luteolysis [9]. The administration of GnRH or its analogs produced a quick increase in the concentration of gonadotropins, which led to the discharge of LH with the end of follicular growth, and lastly, the ovulation [11].

These methods of estrous synchronization lead to a control of the timing of pregnancies and births of our animals in order to facilitate productive management. For this, different practices have been developed to optimize the production and profitability of livestock, such as early pregnancy diagnoses. These groups of techniques allow to identify early and separate pregnant and non-pregnant animals, and act consequently [12]. There are various tools for the early diagnosis of pregnancy in goats; however, not everyone achieves a level of sensitivity, accuracy, speed, security, simplicity and low cost. The development of ultrasonography has become one of the best available technologies for early pregnancy diagnosis [13]. In this way, it not only allows the early checking of the presence of embryo vesicles in the uterus that confirm the pregnancy of the animal, but also offers the opportunity to check the number of fetuses to improve care and feeding if necessary [12].

The B-mode of real-time ultrasonography is a non-invasive, simple, fast and accurate method for the diagnosis of pregnancy in small ruminants on the farm. In addition, it makes possible the study of the pregnancy evolution over time, through the generation of digital images in a gray scale [14].

The ultrasound study has many applications in veterinary medicine, but it is especially interesting in the pregnancy study of animals. One of the most recurrent and researched uses for a long time is the study of the evolution of fetuses throughout pregnancy [15]. The use of B-mode ultrasonography has been established as a good biometric and fetal morphology assessment system. It can correlate several parameters studied with pregnancy age, which allows the prediction of the gestational age of the animal [16]. Several fetal growth parameters have been studied over time, which have shown to have a high correlation with pregnancy age: biparietal diameter, trunk diameter, crown-rump length, eye orbit diameter, cardiac axis length or femur length, among others [17,18,19]. However, the fetal measurements that have been studied may be linked to the breed, so there would be variability in the data between them. This is why it is difficult to extrapolate the data from the studies between different breeds to predict more or less accurately the pregnancy age or the days to delivery (for example, the differences in fetal growth rates between European and Asian races) [20].

Ultrasound resources have developed in order to fit to investigation needs, for instance, Doppler ultrasound [21]. The Doppler effect in ultrasound is based on the fact that when ultrasound waves collide with moving structures, the frequency of the reflected waves is different from the emitted ones, unlike when these waves collide with static surfaces, whose reflection is the same that the emitted ones by the transducer. The Doppler effect is proportional to the speed of movement of the reflecting surface [22]. In the case of ultrasonography, the moving structures that are analyzed are the cells that circulate through the blood vessels. Therefore, there will be positive parameters if the frequency of the reflected waves is higher than those emitted when the blood cells move towards the transducer and negative if the cells move away from the transducer, so the frequency of the reflected waves is lower than those emitted [23].

Doppler can be continuous, without discriminating between depth field, and pulsed-wave, in which the depth of the studied field can be selected. On the other hand, three ways have been described to represent the Doppler signal: Color Doppler allows us to see the change in Doppler frequency in a red-blue color scale depending on the flow direction (red if it approaches the transducer and blue if it moves away). Power Doppler indicates the presence of flow in a structure more sensitively than color Doppler, without providing information on the speed or direction of the flow. Finally, spectral Doppler analyzes the velocity of the vessel flow in a specific period of time, showing a wave that represents the hemodynamics of the blood vessel [24].

Doppler technology in ultrasonography makes it possible to study the vascular system of animals, in addition to some pathological processes such as cancer, edema or injuries recovery. This system is quite quiescent, except for the ovary and pregnancy-related vascular changes [25]. Vessel flows such as uteroplacental arteries, umbilical cord, aorta, caudal vena cava and fetal venous ductus have been investigated by Doppler technology [21]. Ultrasound analysis using Doppler technology has not been extensively studied in the goat species, as well as Doppler parameters in abnormal pregnancies [26]. In small ruminants, Doppler ultrasound study of the waveforms in umbilical artery blood flow may have been performed in order to identify fetuses that may require monitoring during pregnancy or planning for delivery. On the other hand, the umbilical artery pulsations have been widely used as a marker of cardiac functionality as well as fetal health and development [27].

The umbilical cord in small ruminants is described as floating in the amniotic fluid that surrounds the fetus, and it is formed by a bundle of blood vessels made up of two arteries and two umbilical veins arranged in a characteristic spiral position [28]. The umbilical cord connects the growing fetus with the maternal placenta. It transports oxygenated blood and nutrients from the mother through the umbilical veins and removes waste materials and deoxygenated blood for elimination, so the fetal metabolism is assisted [29]. Studies have determined that the vascular system in the umbilical cord adapts to hemodynamic changes to ensure a blood supply for the developing placenta and fetus [26].

In order to study the correct placental function and the well-being of the fetus, a series of parameters have been analyzed by Doppler ultrasonography. These parameters provide information on the optimal course of pregnancy [30]. Initially, the study of blood flow in the umbilical vessels relied on invasive Doppler measurements of the cord. However, the development and refinement of color Doppler ultrasonography techniques has allowed the non-invasive study of blood flow [28]. The main Doppler parameters of blood flow studied are as follows: Arterial Pulse (AP), Peak Systolic Velocity (PSV), End Diastolic Velocity (EDV), Mean Velocity (MV), the Systolic velocity/Diastolic velocity ratio (S/D), Resistance Index (RI) and Pulsatility Index (PI). PSV is the maximum velocity of blood flow through the lumen of the examined vessel in one systole. EDV represents the blood velocity at the end of a cardiac cycle, just before systole. Finally, the MV is the speed of the blood that passes through the interior of the vessels at a specific moment [27,28,31,32,33,34,35].

Because of the problems when analyzing blood flow velocity data, the indices that relate these data such as RI, PI and S/D have been used as more adjusted element [27]. The RI is the ratio between the systolic and diastolic flow that reflects the resistance presented by the blood flow in its course through the lumen of the vessels, assuming the maximum value of the blood flow. RI has been described as being used in those vessels whose blood flow persists during diastole. The PI relates the PSV and the EDV to the MV during the cardiac cycle. It represents the speed of blood flow, suitable for vessels in which the flow is absent during diastole. Both RI and PI were influenced by heart rate and increase if blood perfusion decreases [21,27]. Finally, the S/D ratio is a studied measure of placental resistance in the blood flow circulation [34].

Waveform recordings of the umbilical artery blood flow, such as vascular pulsatility and maternal-fetal vessel resistance, have been part of the routine analysis of fetal well-being, as it is largely related to some alteration in pregnancy [21]. Nevertheless, the accuracy of Doppler measurements can sometimes be limited due to small vessels size or transducer distance [30].

For all these reasons, the aim of this work was to study the evolution of these fetal growth rates and the hemodynamic characteristics of the umbilical artery during pregnancy. For this, in the present study, a group of goats of the M-G breed was synchronized and fetal growth rates and Doppler parameters mentioned were analyzed weekly over pregnancy, from 18- to 125-days post-mating.

## 2. Materials and Methods

### 2.1. Ethics

Through the experiments, animals were handled carefully, avoiding any unnecessary stress. All experiments were performed following relevant guidelines and regulations. The study was carried out in compliance with the ARRIVE guidelines (https://arriveguidelines.org/, accessed on 20 January 2022).

### 2.2. Animals

The animals used in this study belong to Murcian Institute for Agricultural and Environmental Research and Development (IMIDA). IMIDA has a small experimental farm to carry out our research, and all the animals used were born in these facilities. All the goats used for the study were adult goats, which had calved more than once and with an average live weight of 45 kg. The animals have been subject to the sanitary hygienic program established in the Region of Murcia for goats. Initially, 13 healthy primiparous M-G goats were used in the study from February to July of 2022. These goats were located in the Veterinary Teaching Farm of the University of Murcia (Murcia, Spain, 38°0′15.3″ N, 1°10′31.3″ W). Goats were fed with 700 g of maintenance fodder per animal and hay ad libitum during the study. A supplementation of vitamins A, D3 and E (Labhidro^®^ AD3E, Labiana, Barcelona, Spain) was also applied every 15 days. It was administered from 4 to 5 mL per animal at a concentration of: 500,000 IU/mL of vitamin A, 75,000 IU/mL of vitamin D3 and 50 mg/mL of vitamin E. On the other hand, those goats that presented pregnancies of three or more kids were also administered with a supplement of 25 mL of injectable calcium (Labiana, Barcelona, Spain) at a concentration of 216.18 mg/mL.

### 2.3. Synchronization and Mating

In order to facilitate the management of the animals, it was decided to carry out an estrus-synchronization treatment in the goats, so that the pregnant goats in our study could be grouped into one lot. On the first day, vaginal sponges impregnated with 0.03 g of fluorogestone acetate (Sincropart^®^, Ceva, Barcelona, Spain) were implanted using an applicator, leaving the cord of the sponge slightly protruding from the vulva so that it could be easily retrieved. Nine days after having applied the sponges, 2.5 mL of eCG (Foligon^®^, MSD, Madrid, Spain) was administered per goat at the level of the scapula at a concentration of 120 IU/mL. In addition, a dose of PGF2α (Dalmazin^®^, Fatro Ibérica, Barcelona, Spain) of 1 mL per goat (0.075 mg/mL) was also applied, next to the contralateral scapula. Finally, two days later, the sponges were removed from the goats.

The mating of the goats took place by natural mating. The appearance of the estrus between 36 and 48 h after the removal of the vaginal sponges was assumed. For this reason, from the afternoon following the end of the treatment, the bucks were joined with the goats, controlling those that were in estrus. That day was determined as day 0 of pregnancy. The goats were kept with the assigned buck for the following two days, controlling in each case which goats were allowed to be mounted by the bucks each day.

### 2.4. Ultrasonography Study

All ultrasound examinations were carried out at the facilities of the Veterinary Teaching Farm from the University of Murcia. For the ultrasound diagnoses, a General Electric Voluson^®^ 730 PRO scanner was employed (GE Healthcare, Milwaukee, WI, USA). A sectorial abdominal transducer (2–7 MHz, mod. AB2-7) from this ultrasound device was used with an arrangement of the convex-shaped piezoelectric crystals to favor contact with the surface to be explored in the animal, as well as an intracavitary microconvex transducer aimed for endovaginal application (5–9 MHz, mod. IC5-9) (GE Healthcare, Milwaukee, WI, USA). The endovaginal transducer was used exclusively during the first six sessions, from day 18- to day-34 post-mating (dpm). The following two sessions alternated between vaginal and abdominal ultrasound (39–45 dpm). Finally, the abdominal transducer was used weekly in ultrasound examinations, from day 54 dpm, until the last examination carried out in this study (125 dpm).

To perform the ultrasound examinations, animals were placed in a supine position on a stretcher with limbs immobilized and stretched with the head and neck resting on a specific support.

In the first examinations, the endovaginal transducer was used, protected by a cover whose inner end was previously applied with an acoustic gel to allow ultrasound transmission. Before proceeding to introduce it into the goat, Vaseline was applied to lubricate the area and facilitate the introduction of the transducer. For the abdominal ultrasounds, which were carried out a few weeks later, the immobilization procedure for the animals was the same, but in this case the inguinal area close to the mammary gland was shaved with a peeler and cleaned. Subsequently, the acoustic gel was applied to the skin so that, in this way, the air that could exist between the transducer and the skin was eliminated. This way, the transmission of ultrasound through all the structures could be eased. All tests were performed without having to administer any type of sedation to the animals.

#### 2.4.1. Fetal Growth Parameters

B-mode was performed in the ultrasound examinations for an initial search of the different structures, both with the endovaginal and abdominal transducer. This mode was used to take a series of measurements in the embryo/fetus throughout pregnancy: crown-rump length (CRL, from 24 dpm to 61 dpm), trunk diameter (TD, from 24 dpm to 34 dpm), biparietal diameter (BPD, from 28 dpm to 125 dpm) and eye orbit diameter (EOD, from 75 dpm to 125 dpm).

**Figure 1:**
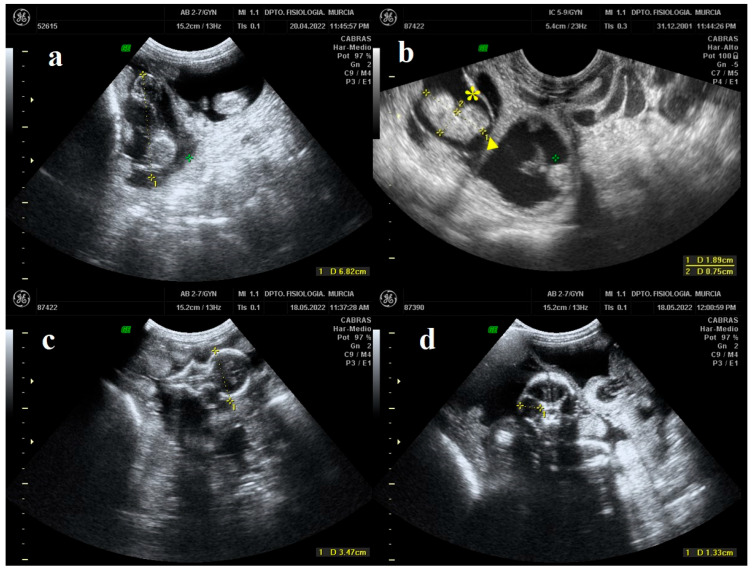
B-mode ultrasound images in which fetal growth indices are measured. (**a**) The yellow line represents the crown-rump length (CRL). (**b**) The yellow line marked with (▶) represents the crown-rump length (CRL) and the one marked with (*) represents the trunk diameter (TD). (**c**) The yellow line represents the biparietal diameter (BPD). (**d**) The yellow line represents the eye orbit diameter (EOD).

Crown-rump length (CRL) has been proven as a common and reliable measure to determine biometric parameters in fetuses during the first third of pregnancy [36]. Measurements were taken from the highest part of the skull to the end of the sacrum (Figure 1a) [37]. On the other hand, the trunk diameter (TD) is the maximum diameter of the fetal body measured from the vertebral column to the ventral portion of the abdomen (Figure 1b) [19].

The biparietal diameter (BPD) is a measurement that is routinely performed in the prenatal examination of pregnancy to estimate the birth date in several goat breeds. For its measurement, a symmetrical view of the fetal skull must be obtained. When looking at the maximum oval-shaped size of the skull (looking at both eye sockets from the front), BPD was measured from one parietal end to the other (Figure 1c) [14]. Finally, the measurement of the eye orbit diameter (EOD) must be taken once it is observed to have a rounded shape at its maximum size, thus measuring the diameter of the circle formed (Figure 1d) [14].

#### 2.4.2. Doppler Ultrasonographic Study of the Blood Flow in the Umbilical Artery

**Figure 2:**
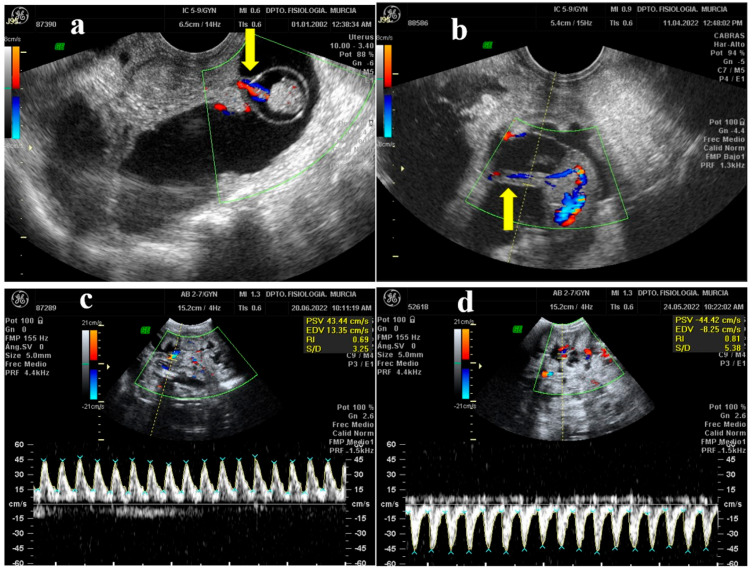
Ultrasound images of the study of the umbilical cord of M-G breed goat fetuses. (**a**) Early identification of the umbilical cord (arrow) of the embryo (32 dpm). (**b**) Color Doppler ultrasound identification of the umbilical cord (arrow), where red colors reflect blood approaching the transducer and blue colors reflect blood moving away. (**c**) Umbilical arterial flow waves by color Doppler and spectral Doppler, where blood flow is directed towards the transducer and shown with positive values above baseline. (**d**) In this image, the flow is moving away from the transducer and is represented by negative values below the baseline. PSV: Peak Systolic Velocity. EDV: End Diastolic Velocity. RI: Resistance Index. S/D: Systolic/Diastolic Ratio.

B-mode enables the capacity to study the moment in which the umbilical cord begins to be glimpsed for the first time (Figure 2a). In the first cord sightings, a small structure was observed next to the embryo, so that it could be located with an elongated or rounded shape as the fetus grew (depending on whether the section we observed was longitudinal or transversal) and floated in the amniotic fluid.

Once the umbilical cord was found thanks to the B mode, it was verified that it was this structure using color Doppler and observing the red-blue color pattern that indicates the presence of positive or negative blood flow (Figure 2b). Next, it was analyzed by spectral Doppler. To begin with, the Doppler caliper was placed over the umbilical artery and the examination began. The graph obtained with the spectral Doppler study on an artery showed the waveform systole and diastole for each cardiac cycle with a characteristic sawtooth shape. However, a vein gave a constant graph since it does not present a pulse pattern. Subsequently, while the graph is displayed, the ultrasound machine transforms the ultrasound into the sound of pulsations. In the case of arteries, this sound was rhythmic and pulsating, while the result of the veins was constant and without a defined pulse.

Firstly, AP measurements were recorded from 34 dpm until the end of the study, at 125 dpm. To do this, the systolic waves in 5 s marked by the spectral Doppler graph were taken and multiplied by 12 to calculate the pulsations per minute of the embryo/fetus. Secondly, umbilical cord blood flow was evaluated semi-quantitatively using Doppler indices. These are relative data obtained from the evaluated PSV and EDV measurements collected by the scanner software by which the MV was found. With these values, RI and PI can be calculated using the following equations: RI = (PSV − EDV)/PSV; PI = (PSV − EDV)/MV. Lastly, the S/D ratio was taken directly from the scanner software.

### 2.5. Experimental Design

A total of 13 goats were synchronized and subsequently inseminated by natural mating at the end of February (24–26 February 2022), with an average temperature and humidity during this month, of 13.1 °C and 62.6%, respectively. At 18 dpm, the first ultrasound was performed to confirm the pregnancy. Next, ultrasounds were performed every 3–4 days during the first six sessions. Subsequently, the ultrasound examinations were carried out once a week throughout the pregnancy (≈150 days), ending with the last test about 25 days before the expected date of deliveries (125 dpm in the last ultrasound) established for the last days of July. In this study, a total of 19 ultrasound sessions have been carried out for each pregnant goat.

### 2.6. Births

Goats were separated from the group once they gave birth. After deliveries, data were collected on the length of gestation of the goats until farrowing, the number of total kids, live births and the alive offspring percentage, as well as sex, birth weight, fetal growth and the color of the coat of the kids.

### 2.7. Statistical Analysis

At the end of each ultrasound examination, the images and videos obtained were collected for the data collection of each session. The collected data were subsequently reviewed, discarding those that did not provide sufficient information or were irregular because they had been taken during maternal and/or fetal movements. Next, the results corresponding to the different parameters analyzed were recorded in Excel sheets for their subsequent statistical treatment.

Statistical analysis was performed using the SPSS v.25 for Windows (IMB SPSS, Chicago, IL, USA). Data were represented by mean ± standard deviation. The different parameters were analyzed using a Shapiro–Wilk normality test. The data showed a normal distribution, so they were analyzed using an analysis of variance (ANOVA). When the data showed significant differences (*p* < 0.05), the values were compared between pairs using a post hoc test (Tukey).

## 3. Results

### 3.1. Synchronizations

After the synchronization treatment, six of the goats started to be in heat around 36 h after the removal of the vaginal sponges, whereas three started between 48 and 60 h and the remaining four started within 72 h after the end of treatment. Once they were covered by the males, pregnancy diagnoses were made by ultrasound 18 days after mating. All goats would be between days 17 and 19 of pregnancy. At this time, vesicles were observed in 12 goats, although some of them were uncertain. At 28 dpm, in a new ultrasound review, the presence of vesicles with embryos inside them was clearly observed in nine of the goats in the study. Three goats were definitely non-pregnant, and embryonic resorption occurred in the last one. Therefore, there were finally a total of nine pregnant goats for the development of the entire experience.

### 3.2. Fetal Growth Indices

**Figure 3:**
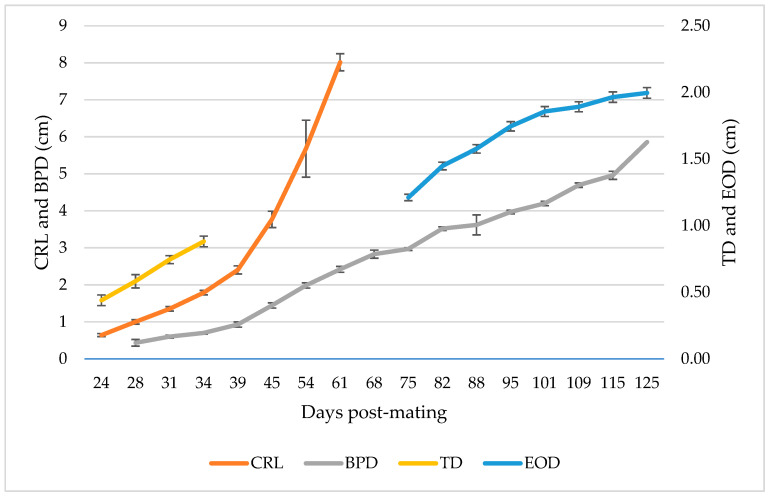
Evolution curves of crown-rump length (CRL) and biparietal diameter (BPD) (left *y*-axis), trunk diameter (TD) and eye orbit diameter (EOD) (right *y*-axis) in total goats under study (*n*: 9). The mean and the standard deviation of the measurements are plotted in relation to the days after mating.

Looking at the graphic representations of the biometric parameters that have been studied throughout pregnancy (Figure 3, Table 1), it is evident how they progressively increase over time, which is something to be expected due to the fetus’ own constant growth within the mother’s womb. The evolution of the measurements taken from the CRL (Figure 3, Table 1) show how they experienced a significant exponential increase from 39 dpm (*p* < 0.01). Furthermore, the TD, despite the small amount of data collected, increased as the days passed (Figure 3), demonstrating the correlation in time of fetal growth. As observed in Table 1, there is a significant difference in the measurements of both parameters, especially as pregnancy time increases. Both CRL and TD were no longer measured due to the difficulty of recording because of the size of the fetus.

On the other hand, the measure that was most studied over time was BPD. As it is shown in Figure 3 and Table 1, BPD data could be collected from day 28 of pregnancy until the last ultrasound on day 125, also observing a progressive increase and significant differences between weeks (*p* < 0.01). Finally, the measurement that took the longest was the EOD, since it could not be clearly visualized until approximately two and a half months of pregnancy, and its analysis was completed with the last ultrasound (Figure 3, Table 1). As is observed in Figure 3, there was also a continuous increase that slowed down towards the end of the pregnancy, presenting in this case also differences between the measurements from one week to another (*p* < 0.01).

### 3.3. Umbilical Artery Blood Flow Doppler Study

**Figure 4:**
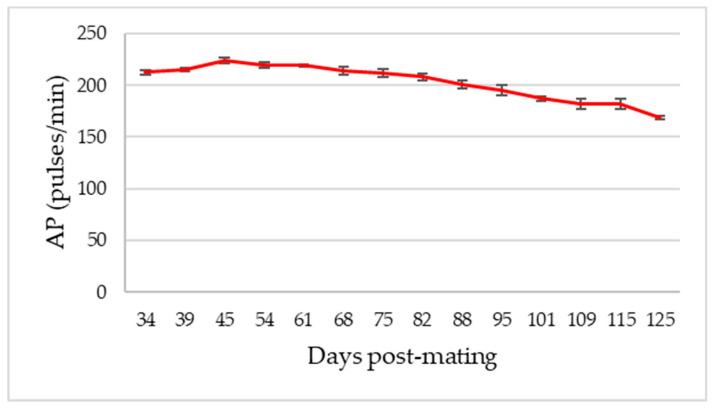
Evolution curve of Arterial Pulse (AP) in total goats under study (*n*: 9). The mean and the standard deviation of the measurements are plotted in relation to the days after mating.

After several ultrasound sessions, it was possible to detect the umbilical cord for the first time in three of the nine pregnant goats between 31 and 32 dpm. For the next session, the umbilical cords could already be observed in eight goats between 33 and 35 dpm. From this moment on, arterial flow was measured using spectral Doppler and AP values were recorded until the end of the study (Figure 4; Table 2).

**Figure 5:**
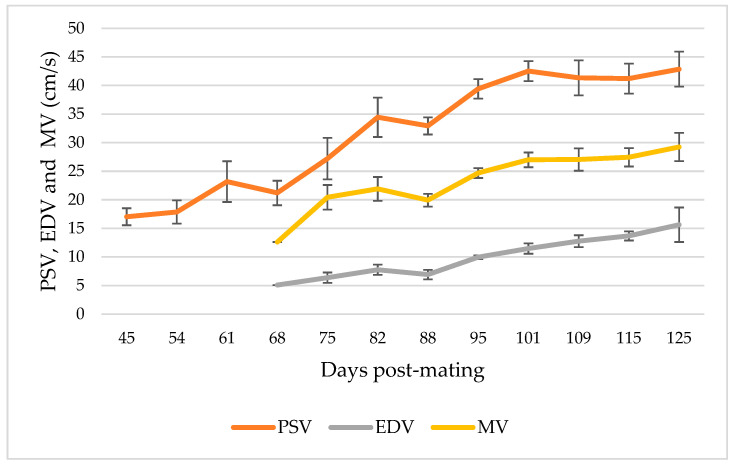
Evolution curves of Peak Systolic Velocity (PSV), End Diastolic Velocity (EDV) and Mean Velocity (MV) in total goats under study (*n*: 9). The mean and the standard deviation of the measurements are plotted in relation to the days after mating.

**Figure 6:**
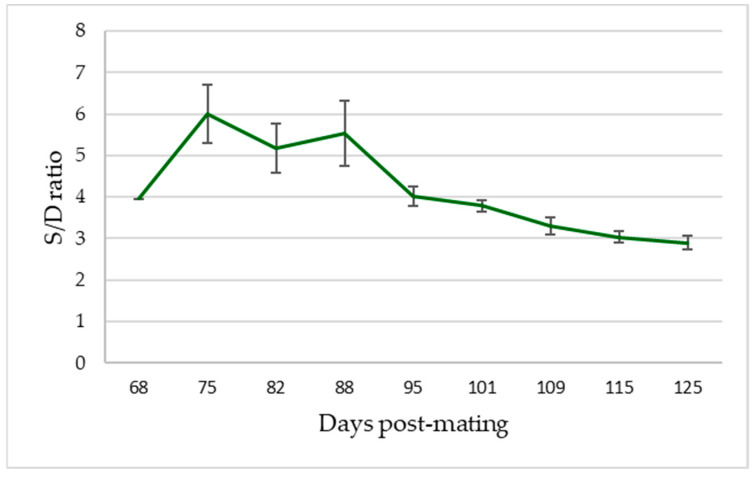
Evolution curve of Systolic/Diastolic ratio (S/D) in total goats under study (*n*: 9). The mean and the standard deviation of the measurements are plotted in relation to the days after mating.

**Figure 7:**
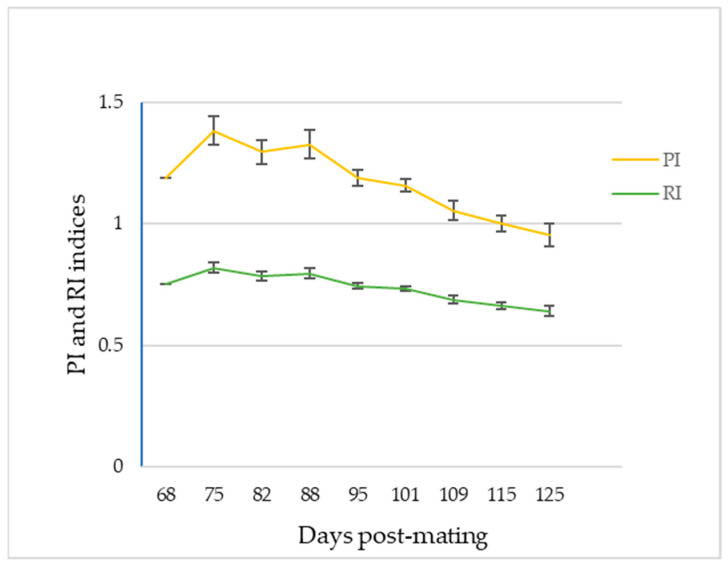
Evolution curves of Pulsatility Index (PI) and Resistance Index (RI) in total goats under study (*n*: 9). The mean and the standard deviation of the measurements are plotted in relation to the days after mating.

From the moment of detection of the umbilical cord until 65–80 dpm, the ratio S/D presented negative values in all the animals and tests performed. This happened even though an adequate AP was recorded with normalized PSV values (after 45 dpm) due to the inaccuracy of the EDV measurements (Figure 5), so the Doppler indices obtained were not conclusive, obtaining RI values > 1. However, from 65 dpm in two goats, 75 dpm in three and 80 dpm in the four remaining animals, and up to the last examination carried out on day 125, all ultrasound controls determined positive S/D ratios (Figure 6, Table 2) and, therefore, representative values of PI and RI (Figure 7, Table 2).

The wave formed by the Doppler when the umbilical artery is analyzed resembles saw teeth, while vein shows a horizontal graph corresponding to the continuous flow of the vein, without pulsations. As is observed from the red and blue pattern of the color Doppler, the vessel measured approaches (red) or moves away (blue) from the transducer. This is translated in the spectral Doppler plot, in which the waves lay above (positive values) or below (negative values) the baseline (Figure 2c,d).

### 3.4. Births

The first goats delivered at 142 days of gestation. Those more advanced to parturition also agreed with the goats that presented a greater number of pregnant fetuses (three and five, respectively). In both goats, two of the kids died, and it is interesting to note that one of the goats with three kids died early. It is likely that the high gestational number was added to the handicap per se, since the delivery was taking place at the end of July in summer season with high average temperature and relative humidity (29.2 °C and 52.5%, respectively). The rest of the goats continued giving birth on the following days without major problems. The six goats with double gestations all gave birth first, without any loss, and finally gave birth to the only goat with a single gestation. Therefore, we see how in multiple pregnancies the births happened earlier than the estimated date compared to twin gestations with two kids or single gestations. All the kids born were weighed and references of sex and coat color were taken. The mean duration of gestations was a few days earlier than expected (approximately 150 days), but looking at the goats individually, the two that gave birth more prematurely coincided with the two that presented a multiple gestation of more than 2 kids. Subsequently, all those that presented twin gestations were successively giving birth. Finally, the last goat to give birth, which is the one with the most days of gestation, was the one with a single gestation. Something similar happens with the average weights of kids at birth (Table 3).

On the other hand, as regards the survival of the kids, it must be said that although the general average of all the goats may be around 80%, the only stillborn kids or those that died around parturition occurred in the two goats with a greater number of pregnant fetuses. Finally, it should be noted that a higher percentage of total females was obtained than males (Table 3).

## 4. Discussion

This is the first time that a detailed study of fetal growth indices and umbilical artery flow rates in fetuses from M-G goats has been performed throughout virtually the entire duration of gestation.

In the first place, the results obtained from estrus synchronization indicated that the protocol used, which is one of the most widely used, left too wide a range in the hours after sponge removal in which the goats were in estrus. It has been described that, after the end of the synchronization treatment, goats usually come into heat at approximately 36 h [38]. However, a broader range was obtained in our study, covering the appearance of estrus from 36 to 72 h after the removal of sponges. In our case, this did not cause a decrease in female fertility rates because each male was left several days with a small group of goats to cover them at the time of the estrus. However, in the case of carrying out artificial insemination at a fixed time, for example, 50 h after sponge removal, as verified by López-Sebastian et al. [39], and as usually happens in the reproductive management of farms, it could be possible to find very early ovulations where the oocyte would be too old when it is inseminated or that the semen remains too long in the reproductive tract of the goat waiting for ovulation.

In our study, a series of fetal growth signal indices were obtained in the M-G goat that could serve as a reference when studying fetal growth, or to identify the gestational age of an animal whose insemination date/covering is unknown. For this, the fetal growth indices CRL, TD, BPD and EOD were analyzed.

CRL measurements were taken between 24 and 61 dpm, as has been reported in other studies in Bulgarian goats [36] and in Egyptian goats [19] in which this parameter was mainly collected around the first trimester of pregnancy, since at this moment it is possible to easily distinguish both ends of the fetus. In the same way, during the remaining two thirds of the gestation, it was not possible to evaluate this measurement due to the difficulty of its complete visualization in the ultrasound image linked to the increase in the size of the fetus. In the case of Karen et al. [19], the ultrasound study of the CRL was extended until 70 days of gestation, but from this moment they could no longer collect any more measurements. Regarding the differences in the values registered between breeds, the studies carried out in Egyptian goats [19,37] and Bulgarian goats [36] showed that there are no large interracial differences for this parameter.

Nevertheless, in the case of TD measurements, while in our case they were analyzed from 24 to 34 dpm, other studies indicate that the period in which this measurement was taken was between days 39 and 85 of pregnancy approximately [20]. This fact is repeated both in the studies reported by the groups of Lee et al. [18] and Karadaev et al. [36], in which these data have been recorded for the TD from 60 to 135 days of gestation in the first case and from 25 to 133 days of gestation in the second, assuming in the latter study that it was possible to take the measurement for a longer time. Observing the differences between breeds, it could be assumed that for the same gestational age there was a greater fetal development in crossbred Saanen [20] and Egyptian [19] goats than in black Korean [18] and Bulgarian goats [36]. However, these data cannot be associated with those obtained in our study, since the measurements were taken mainly on gestational days after ours. Comparing all these data, and despite the statistical association of our results, results of fetal TD measurements in our study should be taken with caution and future analysis of this parameter over a longer gestational period should be carried out.

Regarding the other fetal growth indices, the measurement that was taken in our study for the longest time during pregnancy was BPD, which coincides with other studies in which this parameter was established as one of the most easily detectable during a longer pregnancy period [18,19,40]. Comparing the same days of gestational age, the M-G goat in our study presented similar measurements to Egyptian [37] and Nubian [17] goats, while this parameter was above the average in black Korean [18], Angora [17] and Saanen [16], and below breeds such as Toggenburg [17].

Finally, EOD is a fetal growth index that was measured from 75 to 125 dpm in our study, although the data reported by Karadaev et al. [36] in Bulgarian goats and the group of Yazici et al. [40] in the Saanen breed indicated that EOD measurements could be started from an earlier age (49 and 44 days of pregnancy, respectively), approximately one or two weeks after the measure of BPD began, since it was when the cranial structures could be identified with greater precision. It was noticed that EOD is a good way of fetal measurement, since it was possible to identify the fetal eye orbit for the first time by ultrasonography (which appears as an anechoic circumference in the skull). It was relatively easy to find and measure this orbital structure on successive ultrasound examinations. This is something that was also reported by other groups such as Lee et al. [18], where a high correlation was found between this measure of fetal growth and gestational age.

In our study, the umbilical cord could be observed for the first time in goats between 32 and 35 dpm, which coincides with other studies where it has been identified between 28 and 35 dpm [16,19]. The AP during the first weeks showed unclear values. Firstly, the mean pulse increased, coinciding with the increase in fetal heart rate as the organ developed, and continued by falling after approximately two months of pregnancy. From this moment on, how the beats per minute fell progressively as the fetus grew (*p* < 0.01, Table 2) was observed.

Regarding the results obtained from umbilical artery flow using Doppler ultrasonography, in this study the velocimetric data of both PSV, EDV and MV experienced a significant increase (*p* < 0.01) throughout pregnancy (Table 2); thus, it was stated that the speed of blood flow increases as the fetus develops. The data recorded for PSV initially on day 45 of pregnancy and those for EDV and MV on day 68 agreed with the results reported by Kumar et al. [26], who recorded the first PSV data on day 39 of pregnancy and did not obtain the first EDV data, and therefore MV, until 67 days of gestation. In the records taken by Elmetwally and Meinecke–Tillmann [28], EDV was not measured until the 12th week of pregnancy, for which they proposed that it was able to be associated with the regulation of fetal cardiac cycles and the decrease in heart rate. The explanation for these results could be that, in the early stages of pregnancy, the vascular development at the level of elasticity in the umbilical arteries does not allow a correct recording of blood flow at the diastolic level, and it is from approximately at 2.5 months of pregnancy when these vessels have already acquired enough elasticity so that all the velocimetric parameters and Doppler indices can be determined properly and significantly. Throughout pregnancy, there was a progressive increase in diastolic velocity in the umbilical circulation, leading to a decrease in RI, PI and S/D ratio. This increase in EDV was associated with increased vascularization and decreased vascular resistance in the fetoplacental circulation [35].

As these indicator values of blood flow velocity were not very explanatory by themselves, the different Doppler indices were obtained to correlate and interpret them [27]. Our results with the Doppler indices of RI, PI and S/D indicate that all of them presented a pattern of decrease as the gestation progressed. This correlation was also observed by Irion and Clark [32], who also demonstrated the presence of a statistical relationship between these parameters in their study carried out in sheep fetuses. Firstly, the S/D ratio decreased throughout the gestation, agreeing with the increase in the size of the fetus and the fall in its AP, a fact that was also reflected by Wright and Ridgway [34] in their study in which they demonstrated a correlation between the drop in the S/D ratio and fetal heart rate. The decrease in PI values recorded in our study presented significant differences, as also reflected in the data provided by Martínez–Díaz et al. [27].

The study on the pulsatility of the umbilical artery carried out by Surat and Adamson [35] indicated a correlation between the vascular walls and the vascular resistance of the placenta on this parameter. In case of normal placental resistance, it is the radius of the vessel that is the most important parameter. Therefore, in the earliest stages of gestation from our study, proper EDV recordings were unable to be obtained due to the size of the vascular radius and the elasticity of the artery walls. This is because, as the fetal size increases, the arteries develop greater vascular musculature that decreases their radius and results in a progressive decrease in PI, like that obtained in our study [35]. Such as in other studies [21,27], the RI values in our study decreased progressively and significantly throughout pregnancy. This could be caused by the increased requirements of the fetus due to its rapid growth. In this way, the decrease in the resistance of the umbilical vessels is favored to increase the blood flow of the umbilical cord.

The application of all these analyses of umbilical blood flow can be seen, for instance, in the association that has been demonstrated between the resistance of the umbilical artery determined by Doppler, with intrauterine growth retardation, congenital anomalies and other adverse results in the fetus. The finding of absent or reverse blood flow during umbilical artery systole is a late sign of increased placental vascular resistance [21].

On the other hand, umbilical blood flow is associated with the size of the fetoplacental vascular bed (this one related to fetal size) and with the S/D ratio. This relationship can help us to detect abnormalities in size or function in the fetoplacental vascularization, beyond alterations that occur strictly in the umbilical cord. In the event of a vascular compromise in the fetus, there would be a redistribution of cardiac output focused towards different organs, which could be reflected when analyzing the different Doppler indices of the arterial flow of the umbilical cord [32]. In the human species, the Doppler velocimetric study of the umbilical artery has also been found to be correlated with abnormalities in fetal growth, assuming an effective technique to reduce perinatal deaths associated with fetal growth restriction [41].

Finally, in regard to the births of kids, multiple pregnancies could be related to the greater difficulty in maintaining the pregnancy and, therefore, an increased risk of miscarriage or birth and death postpartum. It should also be considered that the animals were not in the best seasonal conditions, since they were being born at the time of year with higher temperatures. Finally, it would be necessary to indicate the relationship between the days of gestation of the goats and the number of fetuses since it was observed that the greater the number of pregnant children, the shorter the pregnancy, in comparison with those pregnant goats with fewer kids.

## 5. Conclusions

In this study, a series of fetal growth signaling indices have been obtained in the M-G goat that will serve as a reference when studying fetal growth. It can be used to identify the gestational age of goats whose date of insemination/matting is unknown.

The umbilical cord has been identified by ultrasound between 32 and 35 days after mating. In addition, this study has determined that conclusive values for the Doppler parameters and indices cannot be collected until approximately 68 days of pregnancy in M-G goats. This might be influenced by the umbilical artery changes in fetal development.

The evolution of the different Doppler parameters and the umbilical artery pulse showed that velocimetric parameters (PSV, EDV and MV) increased significantly throughout pregnancy in the M-G goat. On the other hand, the values of the arterial pulse, the S/D ratio and the Doppler indices (RI and PI) decreased throughout pregnancy. As has been previously described, there is a relationship between changes that take place while the fetus grows and variations in blood flow of the umbilical artery. Future investigations might shed some light on the study of umbilical blood flow and its significance to the fetus and pregnancy.

## Figures and Tables

**Table 1:** Fetal growth indices measured on different days post-mating (dpm) throughout gestation in total goats under study (*n*: 9). Mean ± Standard Deviation (SD). DPM: days post-mating, CRL: Crown-Rump Length, TD: Trunk Diameter, BPD: Biparietal Diameter, EOD: Eye Orbit Diameter. a–i: Different letters in the same row indicate significant differences.

DPM	24	28	31	34	39	45	54	61	68	75	82	88	95	101	109	115	125	*p*-Value
CRL (cm)	0.64 ± 0.04 a	1.00 ± 0.06 ab	1.35 ± 0.06 ab	1.79 ± 0.06 bc	2.40 ± 0.11 c	3.77 ± 0.22 d	6.42 ± 0.77 e	8.02 ± 0.23 f	-	-	-	-	-	-	-	-	-	<0.01
TD (cm)	0.44 ± 0.04 a	0.58 ± 0.59 ab	0.75 ±0.03 bc	0.88 ± 0.04 c	-	-	-	-	-	-	-	-	-	-	-	-	-	<0.01
BPD (cm)	-	0.44 ± 0.01 a	0.60 ± 0.09 a	0.70 ± 0.04 a	0.93 ± 0.03 a	1.44 ± 0.07 b	1.98 ± 0.07 c	2.42 ± 0.07 d	2.83 ± 0.08 e	2.97 ± 0.11 e	3.52 ± 0.04 f	3.62 ± 0.05 f	3.97 ± 0.27 g	4.20 ± 0.05 g	4.69 ± 0.06 h	4.96 ± 0.06 h	5.86 ± 0.11 i	<0.01
EOD (cm)	-	-	-	-	-	-	-	-	-	1.21 ± 0.03 a	1.45 ± 0.04 b	1.58 ± 0.02 b	1.75 ± 0.04 c	1.86 ± 0.03 cd	1.89 ± 0.03 de	1.96 ± 0.03 de	2.00 ± 0.03 e	<0.01

**Table 2:** Doppler indices of umbilical artery blood flow, measured on different days post-mating (dpm) throughout gestation in total goats under study (*n*: 9). Mean ± Standard Deviation (SD). DPM: days post-mating, AP: Arterial Pulse, PSV: Peak Systolic Velocity, EDV: End Diastolic Velocity, MV: Mean Velocity, PI: Pulsatility Index, RI: Resistance Index, S/D: Systolic/Diastolic ratio. a–f: Different letters in the same row indicate significant differences.

DPM	34	39	45	54	61	68	75	82	88	95	101	109	115	125	*p*-Value
AP (pulses/min)	212.50 ± 2.08 ab	215.56 ± 1.62 ab	224.11 ± 2.66 b	219.38 ± 2.49 b	219.22 ± 1.22 b	214.33 ± 3.67 ab	211.89 ± 4.14 abc	208.22 ± 3.40 abc	201.11 ± 4.04 acd	195.22 ± 5.20 ce	187.00 ± 2.08 de	181.89 ± 5.02 ef	182.00 ± 5.04 ef	168.89 ± 1.93 f	<0.01
PSV (cm/s)	-	-	17.04 ± 1.48 a	17.87 ± 2.03 a	23.19 ± 3.57 ab	21.20 ± 2.14 a	27.21 ± 3.63 abc	34.45 ± 3.45 bcd	32.93 ± 1.50 bcd	39.41 ± 1.71 cd	42.53 ± 1.74 d	41.35 ± 3.06 d	41.21 ± 2.63 d	42.86 ± 3.06 d	<0.01
EDV (cm/s)	-	-	-	-	-	5.10 ± 0.00 ab	6.37 ± 0.91 a	7.76 ± 0.90 a	6.92 ± 0.83 a	9.96 ± 0.31 abc	11.47 ± 0.91 abc	12.76 ± 1.04 bc	13.68 ± 0.80 c	15.64 ± 3.02 c	<0.01
MV (cm/s)	-	-	-	-	-	12.62 ± 0.00 a	20.45 ± 2.16 abc	21.92 ± 2.10 abc	19.93 ± 1.11 ab	24.69 ± 0.87 abc	27.00 ± 1.29 bc	27.06 ± 1.96 bc	27.45 ± 1.61 bc	29.25 ± 2.48 c	<0.01
PI	-	-	-	-	-	1.19 ± 0.00 abc	1.38 ± 0.60 a	1.29 ± 0.05 a	1.31 ± 0.06 a	1.19 ± 0.03 b	1.15 ± 0.03 b	1.06 ± 0.04 bc	1.00 ± 0.03 bc	0.93 ± 0.05 c	<0.01
RI	-	-	-	-	-	0.75 ± 0.00 abc	0.82 ± 0.02 a	0.79 ± 0.02 a	0.79 ± 0.02 a	0.74 ± 0.01 ab	0.73 ± 0.01 abc	0.69 ± 0.02 bcd	0.66 ± 0.01 cd	0.64 ± 0.02 d	<0.01
S/D	-	-	-	-	-	3.95 ± 0.00 abcd	6.01 ± 0.70 a	5.17 ± 0.60 abc	5.53 ± 0.80 ab	4.02 ± 0.20 abcd	3.79 ± 0.14 bc	3.30 ± 0.20 cd	3.04 ± 0.14 d	2.89 ± 0.17 d	<0.01

**Table 3:** Results of births in total goats under study (*n*: 9). ID: Identification number of goat. Mean ± Standard Deviation (SD).

Goat ID	Days of Pregnancy	Total Kids (*n*)	Males (*n*)	Females (*n*)	Kids Born Alive (*n*)	Alive Offspring (%)	Weight (kg)	Coat Color
52,615	149	2	2	0	2	100	2.27	Auburn
							2.18	Auburn
52,618	142	5	2	3	3	60	1.65	Auburn
							1.92	Auburn
							1.75	Auburn
87,271	149	2	0	2	2	100	1.86	Auburn
							1.83	Black
87,289	146	2	0	2	2	100	2.38	Auburn
							2.69	Auburn
87,290	148	2	0	2	2	100	1.87	Auburn
							2.29	Auburn
87,390	142	3	2	0	1	33.33	2.05	Auburn
87,422	147	2	0	2	2	100	2.13	Auburn
							2.38	Auburn
88,573	150	2	1	1	2	100	2.37	Auburn
							2.5	Black
88,586	152	1	1	0	1	100	3.13	Black
Mean ± SD	147.22 ± 1.14	2.33 ± 0.37	0.89 ± 0.31	1.33 ± 0.37	1.89 ± 0.20	80.95 ± 8.15	2.19 ± 0.09	-

## Data Availability

The original contributions presented in the study are included in the article, and further inquiries can be directed to the corresponding author.
